# Protease Responsive Essential Amino-Acid Based Nanocarriers for Near-Infrared Imaging

**DOI:** 10.1038/s41598-019-56871-4

**Published:** 2019-12-30

**Authors:** Anshu Kumari, Kalpana Kumari, Sharad Gupta

**Affiliations:** 1 Discipline of Biosciences and Biomedical Engineering, Indian Institute of Technology Indore, Simrol, Indore, Madhya Pradesh 453552 India; 2 Metallurgical Engineering and Material Science, Indian Institute of Technology Indore, Simrol, Indore, Madhya Pradesh 453552 India

**Keywords:** Molecular self-assembly, Nanoparticles

## Abstract

Delivery of the theranostic agents with effective concentration to the desired sites inside the body is a major challenge in disease management. Nanotechnology has gained attention for the delivery of theranostic agents to the targeted location. The use of essential amino-acid based homopolymers for the synthesis of biocompatible and biodegradable nanoparticles (NPs) could serve as a nanocarrier for delivery applications. In this study, poly-l-lysine (PLL) and salts were used to fabricate the NPs for the delivery of exogenous contrast agents. Here, indocyanine green (ICG) was encapsulated within these NPs, and a simple two-step green chemistry-based self-assembly process was used for the fabrication. The morphological and biochemical characterizations confirm the formation of ICG encapsulating spherical PLL NPs with an average diameter of ~225 nm. Further, a detailed study has been carried out to understand the role of constituents in the assembly mechanism of PLL NPs. Our results show a controlled release of the ICG from PLL NPs in the presence of the proteolytic enzyme. *In-vitro* cellular studies suggest that the PLL NPs were readily taken up by the cells showing their superior delivery efficiency of ICG in comparison to the free-form of the ICG.

## Introduction

Cancer is one of the deadliest diseases worldwide. An early-stage cancer diagnosis is an extremely crucial step towards successful treatment and to improve patient survival rate^1^. Nowadays, several dyes and drugs are being used for cancer diagnosis and therapy^2–8^. However, the delivery of these theranostic (drug/dye) molecules to the specific location in the body with effective concentration is a challenge. Nanobiotechnology has played an important role in site-specific drug delivery applications^9–12^. This technology improved the efficiency of theranostic agents by simultaneously increasing molecule half-life, the effective concentration at the specific location, and reducing the side-effects^13,14^. Recently, various nanoparticles, such as metal nanoparticles (NPs), polymer-based NPs, and liposomes, *etc*. have been developed as drug carriers^15–24^. However, the major constraints of most of these NPs are their poor biocompatibility, non-biodegradability, and long and short-term tissue toxicity^25–28^. As a result, considerable interest has been shown to fabricate the NPs using biomacromolecules like lipids, amino-acids, and proteins for the delivery of drugs and contrast agents^29–32^.

The utilization of essential amino-acid based homopolymers could be an alternative for the fabrication of the biocompatible and biodegradable nanocarriers for delivery applications. Poly-l-lysine (PLL) is a homopolymer of an essential amino-acid, lysine, which plays a vital role in the body^33^. The human body utilizes it for bone development, protein synthesis, and the production of collagen by promoting absorption of calcium^34,35^. In addition, it helps in the production of various hormones, antibodies and enzymes, and tissue repairment^36^. Moreover, PLL is a cationic homopeptide, generally being used for cell adhesion in the cell culture plates from the last four decades^37^. Recently, PLL has gained the significant attention of the scientific community in the domain of gene therapy and surface coating of metal particles^38–43^. Also, PLL composite with metals gained interest as a potential carrier for drug delivery. To overcome the limitations of the theranostic molecules, Wu *et al*. encapsulated molecule in triblock copolymer micelles of poly(ethylene glycol)-b-poly(L-lysine)-b-poly(L-leucine) (PEG-PLL-PLLeu) and Wang *et al*. used a composite of PLL with Pt(II)-porphyrins^44,45^. However, these composites of the PLL are not completely biodegradable, and their fabrication process requires organic solvents, which might have toxic effects. In this article, we introduce a two-step green chemistry-based method for the fabrication of biodegradable nanocarriers from PLL in an aqueous medium.

For the proof of concept, indocyanine green (ICG) was encapsulated within PLL NPs. ICG is a US Food and Drug Administration (FDA) approved near-infrared (NIR) fluorescent dye used for various biomedicalapplications^46–49^. Despite its several applications, ICG has not been used to the fullest for deep tissue imaging in its free-form. The free-form of the ICG suffers from off-site delivery, concentration-dependent aggregation, and poor photostability in the aqueous solution^50,51^. These limitations restrain the application of ICG to be used as a NIR active exogenous contrast agent for bioimaging. An appropriate approach to address these shortcomings would be to encapsulate free ICG within nanocarriers. In the present study, we demonstrate a simple green chemistry-based new formulation of ICG within PLL NPs, which has potential applications in NIR bioimaging. In this formulation, ICG was loaded within the PLL NPs via a two-step self-assembly method. It is pertinent to note that the assembly of PLL NPs was accomplished in an aqueous medium by mixing of the essential amino-acid homopeptide, ICG, and multivalent anionic salts. All components used for the fabrication were nontoxic in nature. The systematic mixing of all the components results in the formation of stable and monodisperse NIR active PLL NPs. These nanoparticles were aimed to have protease responsive nature in order to efficiently deliver the cargo within the cells. The design rationale of protease responsive NPs lies in the fact that the proteolytic enzymes are present in the lysosomal compartment, which hydrolyze these NPs and triggers the release of encapsulated cargo^52–54^. The advantage of PLL NPs is that the presence of the proteolytic enzymes will regulate the cargo release. Therefore, PLL NPs would only release ICG when taken up into the cells via endocytosis and trafficked to lysosomes, where proteolytic enzymes degrade PLL NPs. The degradation byproducts of these NPs are the fragments of lysine, salts, and the free-form of ICG. These byproducts would be utilized by the cells without showing any toxic effect, whereas the ICG would be used to stain the cells for NIR bioimaging. In comparison with other conventional NPs, the PLL NPs are completely biodegradable and biocompatible. The *in-vitro* cellular uptake study result shows that PLL NPs incubated cells had significantly higher NIR fluorescence emission in comparison to the free-form of ICG. To the best of our knowledge, this is the first report on the green chemistry-based fabrication of ICG encapsulating protease responsive PLL NPs for NIR bioimaging. In addition to ICG, these NPs could also be used for the encapsulation of various theranostic agents such as hydrophobic anticancer molecules, chemotherapeutic drugs, *etc*. for targeted drug delivery.

## Materials and Methods

### Materials

PLL (Molecular weight = 120 kDa, ~574 lysine unit, one HBr per lysine residual) was procured from Polysciences (Warrington, PA, USA). Trisodium citrate dihydrate was procured from the Merck (Darmstadt, Germany). Sodium phosphate dibasic heptahydrate, fluoromount mounting media, and ICG were purchased from the Sigma Aldrich (St. Louis, MO, USA) and were used as received without further purification. Stock solutions of the ICG and salts were prepared in de-ionized (DI) water (18.2 MΩ Millipore, Sartorius system). All the prepared stock solutions were stored at 4 °C. Cancerous cells were procured from the National Centre for Cell Science (NCCS) Pune, India. Fetal Bovine Serum (FBS), Dulbecco’s Modified Eagle Medium (DMEM), 0.25% trypsin-1 mM ethylenediaminetetraacetic acid (EDTA), penicillin-streptomycin, and 2.5% trypsin without phenol red were purchased from Gibco (Thermo Fisher Scientific Inc., India). Colorimetric assay (MTT, (3-(4, 5-dimethylthiazol-2-yl)-2, 5-diphenyltetrazolium bromide)) was procured from Himedia chemicals (India). For nucleus staining, DAPI (2-(4-amidinophenyl) indole-6-carboxamide-dihydrochloride) was procured from the Tokyo Chemical Industry (TCI, India).

### Synthesis of ICG loaded PLL NPs

**Figure 1:**
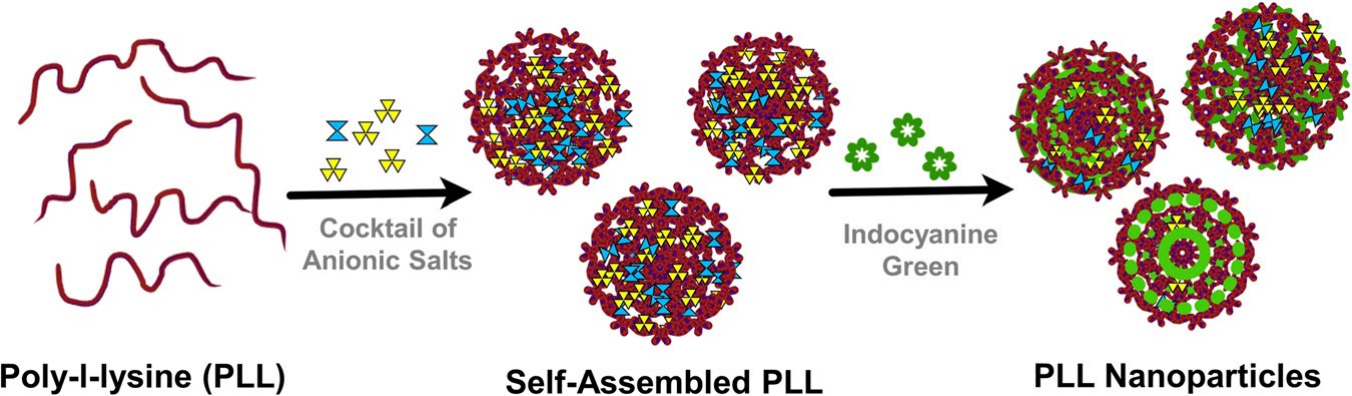
Schematic diagram representing fabrication process of poly-l-lysine nanoparticles (PLL NPs) encapsulation indocyanine green (ICG); mixing of cationic polypeptide with a cocktail of the anionic salts namely sodium phosphate and trisodium citrate results in NPs formation then ICG was added and aged for 30 mins which results in encapsulation of ICG with in PLL NPs.

A two-step self-assembly method has been used for the synthesis of the protease responsive PLL NPs. Figure 1 displays the schematic illustration of the fabrication process, where ICG was encapsulated within PLL NPs. Concisely, 20 µL of PLL solution (3 mg/mL) was taken in a 1.5 mL microcentrifuge tube, which was gently mixed with the cocktail of salts, i.e., 13.2 µL tri-sodium citrate (0.01 M) and 2 µL disodium phosphate heptahydrate (0.01 M) for 10 seconds. The initial clear solution turns into a turbid solution, which indicates the initiation of the nucleation fabrication. In the next step, 200 μL of ICG was added to the polymer/salt colloidal solution. This reaction was completed by adding one mL of DI water to the colloidal suspension. Fabricated PLL NPs were then aged for 30 min at 4 °C. The molar charge ratio (MCR) of the solution was set to be 2 for the fabrication of stable and monodisperse PLL NPs. The MCR was calculated by Eq. (1).

1 $${\rm{MCR}}=\frac{Total\,anionic\,charge\,of\,the\,system}{Total\,cationic\,charge\,of\,polypeptide}$$

After 30 min of aging, the PLL NPs suspension was differentially centrifuged three times at 6,500 rotations per min (rpm) for 1 min, followed by centrifugation at 6,000 rpm for 30 min, and 5,500 rpm for 60 min to get monodisperse PLL NPs.

### Morphological characterization

To assess the morphology of nanoparticles, PLL NPs were imaged by scanning electron microscope (SEM) (Supra-55, Carl Zeiss Sigma Series). For electron microscopy imaging PLL NPs were freeze-dried using a lyophilizer (model: Alpha 1–2 LD plus, Labmate). Following freeze-drying, the carbon tape was mounted on an aluminum stub, and the dried NPs were placed on carbon tape. Before visualization in SEM, the PLL NPs were sputter-coated with gold by direct current (DC) sputter coater (Q-150RES, Quorem) for better electron conductivity. For imaging, secondary electron images were taken in the range of 2 to 5 kV of the electron beam with a working distance of ~10 mm. The FESEM images were analyzed using IMAGE J software (National Institutes of Health, Bethesda, USA)^55^.

### Dynamic light scattering analysis

The zeta (ζ) potential and hydrodynamic diameter of the PLL NPs were measured using dynamic light scattering (DLS) spectroscopy. The DLS instrument (Model-NanoPlus-3, Micromeritics) was equipped with a 660 nm diode laser. The scattering light collection geometry was at 45° for ζ potential and 175° for particle size measurements. The cumulant method was used for the calculation of the intensity averaged hydrodynamic diameter and polydispersity index (PDI). All the samples were measured at 25 °C in DI water three times, and their average is reported.

### Spectroscopic analysis

Steady-state absorption was measured in the UV-Vis-NIR wavelength range using a spectrophotometer (Lambda-35, Perkin Elmer). The absorption spectra were collected at a 480 nm/min scan rate and with a 1 nm slit width. The fluorescence emission spectra were measured using a spectrofluorometer (FL3-21, Jobin Yvon, Horiba). A Xenon lamp of 400 Watts was used as an excitation source for all the measurements in the spectrofluorometer. Fluorescence measurements were carried out using right-angle geometry with 5 nm excitation and 5 nm emission slit width. Emission spectral data were collected using inbuilt fluorescence software, and origin 8.0 was used for further analysis. The optical measurements were carried out in a Hellma 10 × 2 mm quartz cuvette (Muellheim/Baden, Germany).

### Circular dichroism analysis

Circular dichroism (CD) spectroscopic studies were recorded by using a JASCO spectropolarimeter (Model-J-815, Tokyo, Japan). All measurements were done in a Hellma 10 × 2 mm quartz cuvette with a slit width of one nm at a rate of 20 nm per min. All CD spectra were obtained after averaging three scans and were corrected by subtracting the blank spectrum. During measurements temperature was maintained at 25 °C using a Peltier temperature controller.

### *In-silico* studies

Automated docking (AutoDock 4.2, graphical user interface) was used for macromolecule (polypeptide, PLL) and ligand (ICG) docking^56^. ChemDraw Ultra 12 and Chem3D Pro 12 were used to generate the three-dimensional structure of PLL and ICG. Further, the energy of the generated structure was minimized using Density Functional Theory (DFT). For docking, preparation of the target macromolecule with AutoDock Tools (ADT) involved the following steps. Initially, the addition of polar hydrogen atoms followed by partial charge correction. In the final step, the Gasteiger charges of each atom of macromolecule were calculated. In addition, a grid of 40 Å × 40 Å × 40 Å was positioned around the macromolecule with a grid spacing of 1 Å. Lamarckian Genetic Algorithm Local Search (GALS) method was used for the docking calculations, which consist of 25 million energy evaluations (ga_num_evals). The maximum number of iterations per local search was set to 100, and all other parameters were set to defaults with the maximum number of generation (ga_num_generation) is 27,000. The 6 and 7 docking results were clustered based on the free energy of binding and hydrogen bond interactions. Finally, UCSF-Chimera visualization software was used to visualize and analyses the integration of the PLL and ICG^57^.

### Estimation of encapsulation efficiency of ICG

The loading efficiency of the ICG within PLL NPs was measured by Eq. 2. The synthesized NPs were collected after centrifugation and were exposed to dimethyl sulfoxide (DMSO), which results in the instant release of ICG from NPs. Further, the absorption spectra of disintegrated PLL NPs were recorded, and the ICG concentration within these NPs was calculated from the calibration curve.

2 $${\rm{EE}}( \% )=(\frac{{\rm{The}}\,{\rm{concentration}}\,{\rm{of}}\,{\rm{ICG}}\,{\rm{after}}\,{\rm{disassembly}}\,{\rm{of}}\,{\rm{PLL}}\,{\rm{NPs}}}{{\rm{The}}\,{\rm{total}}\,{\rm{concentration}}\,{\rm{of}}\,{\rm{ICG}}\,{\rm{used}}\,{\rm{for}}\,{\rm{the}}\,{\rm{PLL}}\,{\rm{NPs}}\,{\rm{synthesis}}})\ast 100$$

The calibration curve of free ICG in DMSO is shown in Supplementary Fig. S4.

### *In-vitro* release study

The *in-vitro* release profile of the ICG from PLL NPs was studied using the dispersion method in the presence of the proteolytic enzyme^54^. The release profile of ICG form PLL NPs was measured in the presence of a proteolytic enzyme (trypsin) for 24 hours (h). The sample was prepared and aliquoted in five 1.5 mL centrifuge tubes and then centrifuged for the collection of all PLL NPs. Further, 1 mL trypsin without phenol red was added to the pellet of PLL NPs at a concentration of 1 mg/mL and incubated at 37 °C. After each incubation time point, samples were centrifuged, and the absorbance of the supernatant was measured at 778 nm using a spectrophotometer. The amount of ICG released from PLL NPs was calculated using Eq. 3.

3 $${\rm{Cumulative}}\,{\rm{release}}\,( \% )=\{\frac{{\rm{Absorption}}\,{\rm{intensity}}\,{\rm{of}}\,{\rm{supernatant}}\,{\rm{after}}\,{\rm{every}}\,{\rm{time}}\,{\rm{point}}}{{\rm{Absorption}}\,{\rm{Intensity}}\,{\rm{of}}\,{\rm{free}}\,{\rm{ICG}}}\}\ast 100$$

### Cell culture

Cervical cancer cell line (HeLa) was used for all the experiments carried out in this article. HeLa cells were cultured and grown in DMEM media supplemented with 100 units/mL penicillin-streptomycin and 10% FBS. HeLa cells were cultured and incubated at 37 °C supplemented with 5% carbon dioxide (CO_2_). When cells reach ~80% confluency, they were subcultured after detaching with 0.25% trypsin-EDTA.

### Cell viability assay

HeLa cells were seeded in 96 well plates in triplicate (5000 cells/well) in complete DMEM media. After 24 h, the treatment of freshly prepared PLL NP at different concentrations (2, 5, 10, 20, and 50 μL) was added to cells and samples were allowed to incubate for 24 h at 37 °C. The cells without NPs were used as a positive control, and cells treated with 0.3% Triton-X was used as a negative control. The viability of PLL NPs was evaluated by a standard MTT assay. After 24 h, the media was aspirated, and freshly prepared media containing MTT at a concentration of 0.5 mg/mL was added to each well. After incubation for 4 h, the media was carefully removed, and the insoluble formazan crystals synthesized by the viable cells were dissolved by adding 200 µL dimethyl sulfoxide (DMSO) per well. The absorbance of the synthesized formazan was quantified using a UV-Vis microplate spectrometer (SynergyH1, BioTek) at 570 nm. The cellular viability was calculated as a percentage decrease in viability with respect to untreated (control) cells.

### Cellular uptake study

NIR imaging was carried out to evaluate the uptake of PLL NPs by the cancerous cells. HeLa cells were cultured and seeded onto coverslips in a 6 well plate (~2 × 10^5^ cells/well) and were allowed to adhere for 24 h. For the experiment, the media was removed and replaced with two different concentrations of PLL NPs (20 μL and 50 μL), free ICG (3 µM) mixed in the cell culture media and plain media control (untreated cells) were incubated for 4 h in an incubator at 37 °C with 5% CO_2_ supply. Following incubation, the cell culture media was removed, and cells were washed with 1x PBS thrice, and cell nuclei were stained with DAPI. Cell fixation was done with 4% of paraformaldehyde for 30 min at 4 °C, and slides were prepared with the help of mounting media for visualization. The NIR fluorescence imaging of HeLa cells was performed on a Nikon Eclipse Ti-U inverted microscope system. The cells were excited by mercury and xenon lamps for DAPI and ICG excitation, respectively. The exposure time to capture NIR images was 10 seconds, and the exposure time for DAPI images was 0.5 seconds.

## Result and Discussion

In this work, the fabrication of protease responsive PLL NPs via a green chemistry-based approach is reported. In this formulation, a simple two-step self-assembly process was used to encapsulate ICG within the homopolymer of lysine (PLL) NPs for NIR bioimaging. It is noteworthy that in this formulation, no toxic or organic chemical reagents were used. Further, this formulation also protected ICG from optical degradation and could be used as an exogenous contrast agent for NIR bioimaging. The morphological, physiochemical, and biological studies were carried out on these NPs to test their effectiveness to be used as NIR fluorescence imaging contrast agent, in comparison to the free formulation of ICG.

### Biophysical characterization

**Figure 2:**
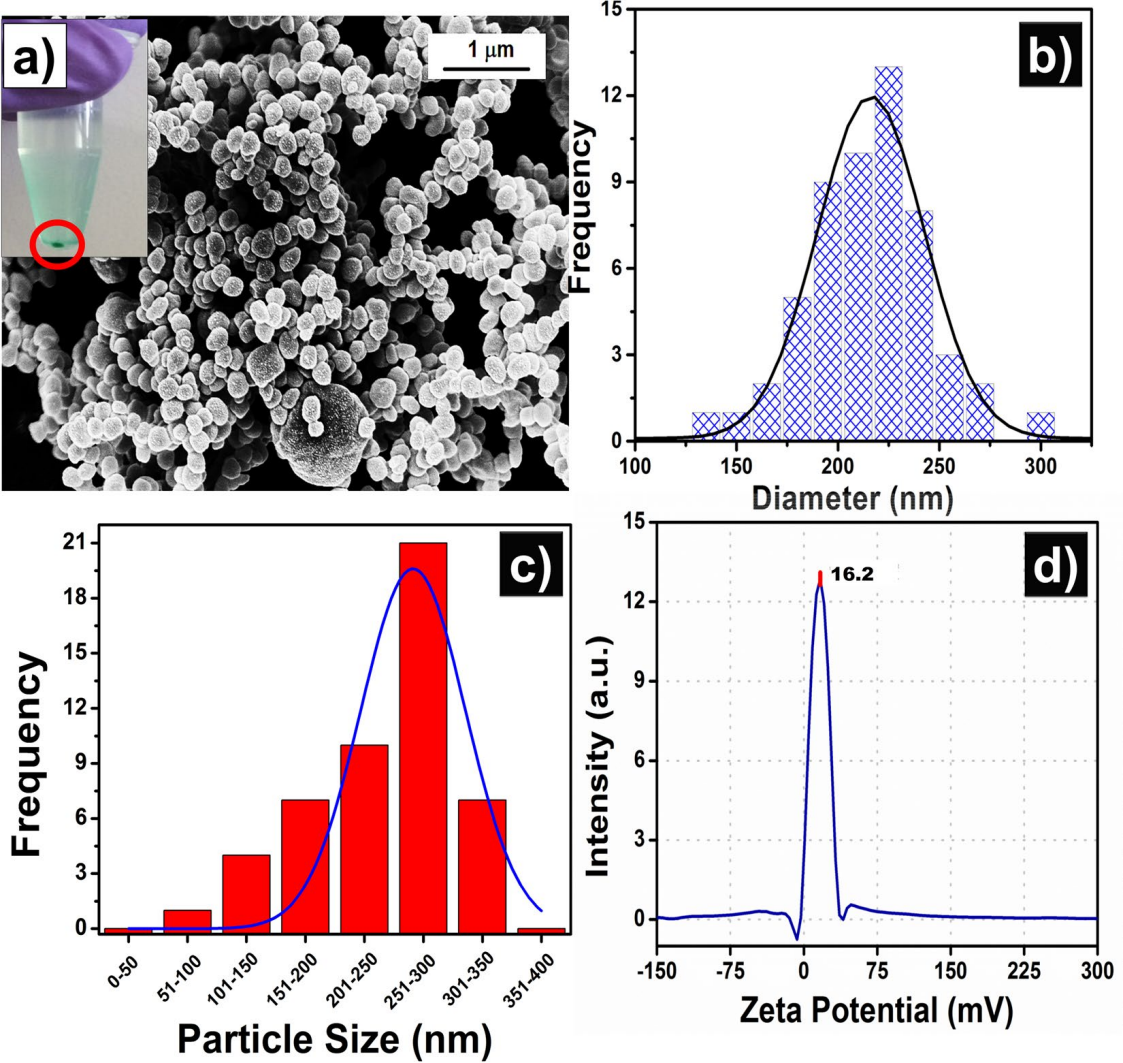
Morphological characterization of poly-l-lysine nanoparticles (PLL NPs). (**a**) Scanning electron microscopy (SEM) image of PLL NPs, the inset shows the green pellet of PLL NPs containing ICG. (**b**) Shows the frequency distribution of PLL NPs of SEM images calculated by IMAGE J software. (**c**) Dynamic light scattering (DLS) measurement of synthesized PLL NPs shows the average hydrodynamic diameter ranging between 251–300 nm. (**d**) Zeta (ζ) potential of PLL NPs.

The self-assembly method has been used to prepare polypeptide nanostructures for various applications^58^. Here, PLL was used for the preparation of the protease responsive NIR active NPs via the self-assembly method. The complete fabrication process of PLL NPs is shown in Supplementary Fig. S1. Briefly, PLL, salts, and free ICG were mixed systematically, which results in the formation of the NIR active PLL NPs, as shown in Fig. 2. The morphology, size, and size distribution of the PLL NPs were determined by SEM. As shown in Fig. 2(a), the fabricated PLL NPs were spherical in shape without any aggregation. The NPs were subjected to the freeze-drying step before SEM imaging, but no damage was observed in the NPs, this suggests the robustness of the NPs. The inset of Fig. 2(a) shows the image of the NP pellet, which was used for SEM characterization. The size-frequency distribution of PLL NPs was calculated from FESEM images using IMAGE J software. As shown in Fig. 2(b) the average size of PLL NPs was found to be ~225 nm.

Hydrodynamic diameter and the ζ potential of the PLL NPs in aqueous solution were measured using DLS, and the results are shown in Fig. 2(c,d). The samples were sonicated for one min before DLS measurements to avoid any possible aggregation. As shown in Fig. 2(c), the hydrodynamic diameter of PLL NPs is in the range of 251 nm to 300 nm. The PLL NPs also exhibit a high degree of monodispersity with a PDI of 0.31. The ζ potential is a function of the particle surface charge and also indicates the stability of the NP. The larger magnitude of ζ potential suggests the formation of a stable nanostructure system without aggregation^59^. As shown in Fig. 2(d), the PLL NPs are positively charged with +16.2 mV ζ potential in DI water, which suggests that these particles are stable in the DI water without any aggregation.

### Effect of pH value and molar charge ratio (MCR) on particles size

**Figure 3:**
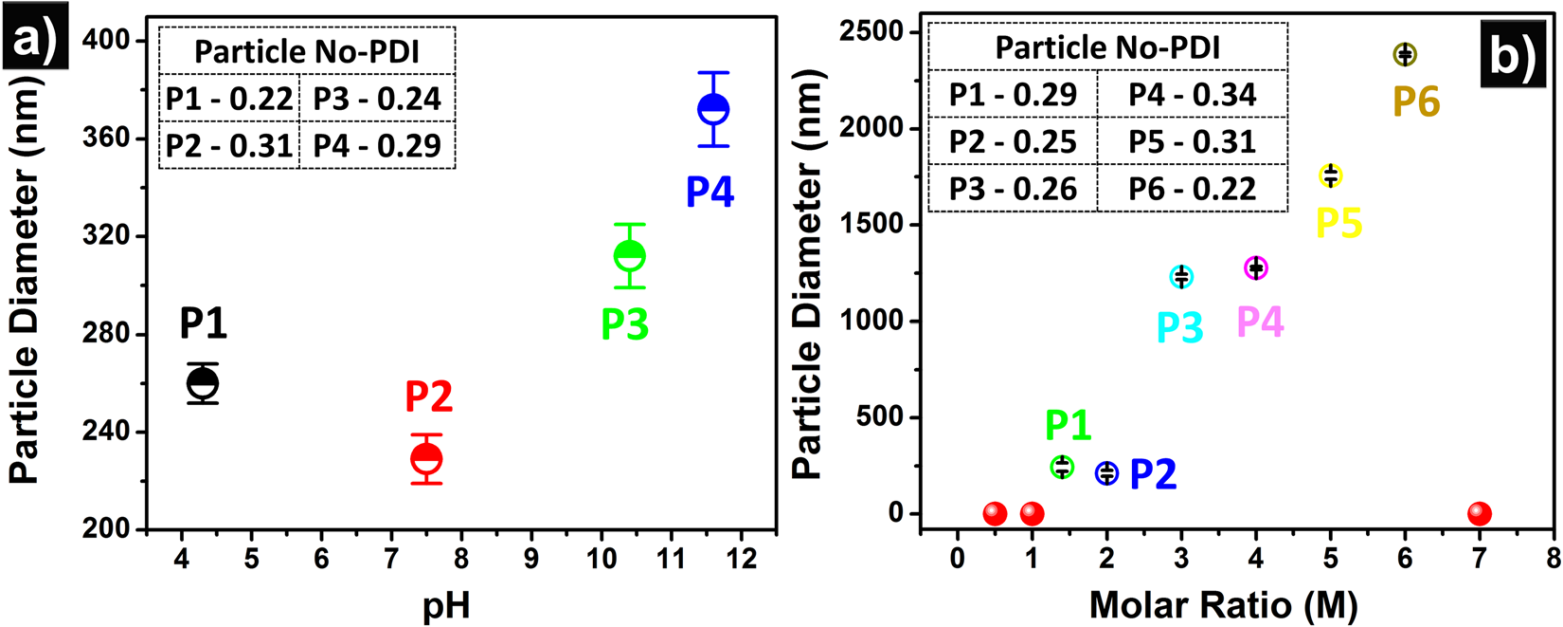
Variation in PLL NPs size due to change in salt pH and molar charge ratio (MCR) (**a**) effect of pH on particle size (**b**) effect of MCR on particle size inset of both the figure shows the particle polydispersity index (PDI).

The self-assembly process for making nanostructure depends on several factors, such as the concentration of the constituents, pH of the salt solution, incubation time, *etc*. The condition for PLL NPs fabrication was optimized by varying multiple parameters. Here, we studied the effect of variation of the pH value and MCR of the system on the size of PLL NPs (Fig. 3). The samples were prepared by varying the pH value of the salts (pH value ranging from 4.5 to 12). The particle size measurements were carried out by using DLS. As shown in Fig. 3(a), the diameter of the PLL NPs varies with a change in the pH values of the salts. The optimal pH value of the salt solution was found to be ~7 (neutral pH) for the fabrication, which produced perfectly spherical PLL NPs of ~229 nm diameter, and the PDI value of 0.31 shows the monodispersity of the formed NPs. The basic pH value of the salt solution (such as 10.4 and 11.6) produced larger size NPs with a particle diameter of more than ~312 nm with lower PDI values due to the variation of charge ratio of lysine at this pH. On the other hand, the acidic pH value resulted in the formation of a bit larger PLL NPs with ~260 nm diameter and 0.22 PDI value. This experiment suggests that the neutral pH value of salt solution would give the smallest and most uniform size NPs without aggregation. In addition to the DLS measurement, the SEM images of the synthesized NPs at different pH values are shown in Supplementary Fig. S2.

The MCR value of the system also plays a crucial role in the fabrication of the NPs. To study the effect of MCR on the size and polydispersity of the NPs, the NPs were fabricated at following MCR values, i.e., 0.5, 1, 1.4, 2, 3, 4, 5, 6, and 7. The size of the NPs was measured by DLS at different MCR values. It was observed that the NPs were not formed when the MCR of the solution was either ≤1 or ≥7 (as shown in solid red circles Fig. 3(b)). However, the stable NPs were formed when the MCR was within the range of 1 to 7. Different values of the MCR resulted in the different sizes of PLL NPs. The smallest size of the (~222 nm) particles was obtained when the MCR was set to be 2. Further, it was observed that with increasing MCR, there is a linear increase in the particle size. For all the different values of MCR, the PDI was below 0.35, which shows the monodisperse nature of the PLL NPs in aqueous solution. The SEM images of the synthesized NPs at different values of MCR are also shown in Supplementary Fig. S3.

### Spectroscopic characterization

**Figure 4:**
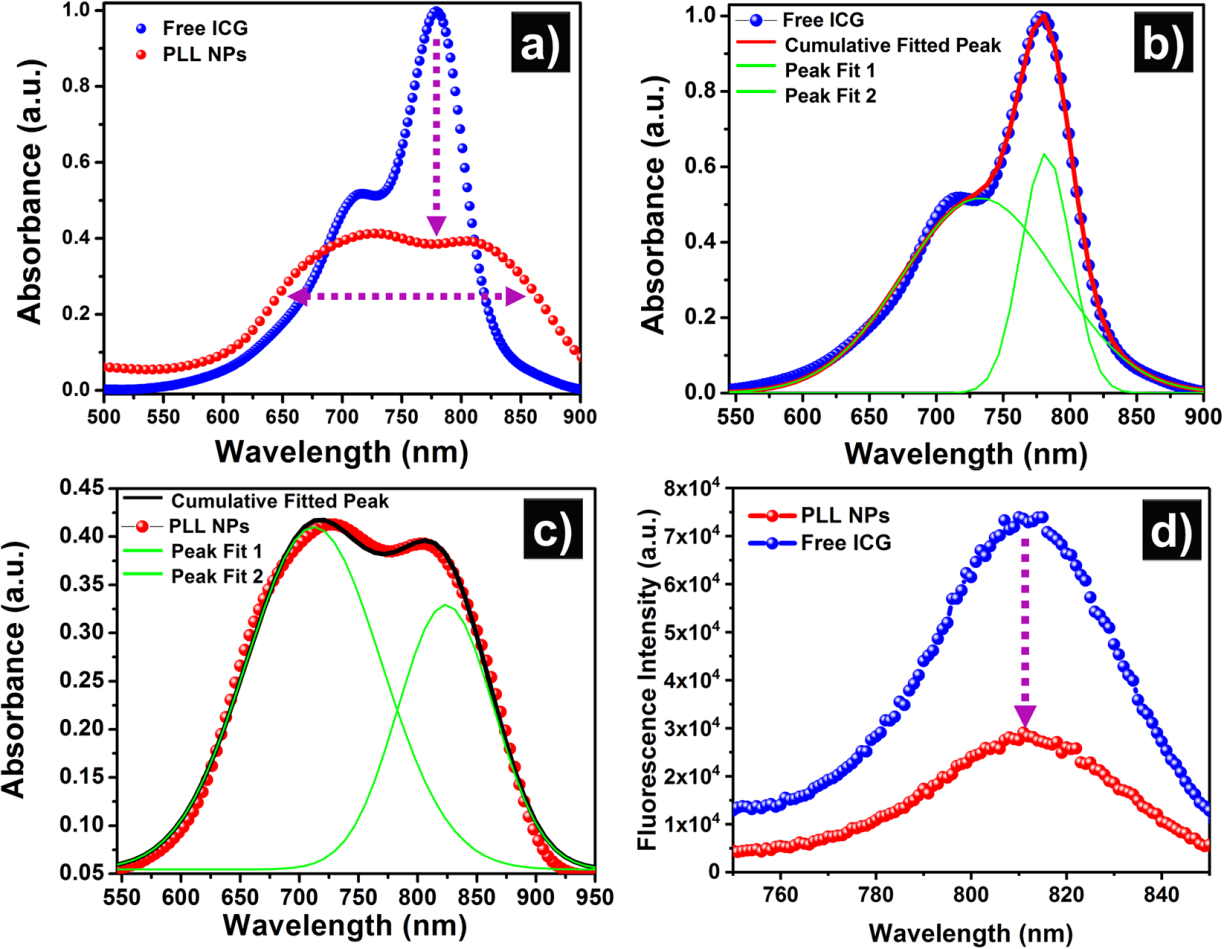
Biochemical characterizations of free indocyanine green (ICG) and poly-l-lysine nanoparticles (PLL NPs). (**a**) Absorption spectra of free ICG and PLL NPs. (**b**) Absorption spectra curve fitting of free ICG with R^2^ = 99.8%. (**c**) Absorption spectra curve fitting of PLL NPs with R^2^ = 99.4%. (d) Emission spectra of free ICG and PLL NPs.

The optical absorption and fluorescence emission spectra of free ICG and NIR active PLL NPs are shown in Fig. 4. Significant reduction and noticeable broadening of the absorption peaks was observed in case of PLL NPs when compared with the free ICG, as shown in Fig. 4(a). The absorption spectrum of the free ICG shows a monomeric peak centered at 782 nm and a smaller aggregated vibronic shoulder peak at 732 nm^60^. A Gaussian peak fitting was used to differentiate the contribution of the monomeric and aggregated forms of ICG molecules in its free and encapsulated form (PLL NPs), as shown in Fig. 4(b,c). Further, the peak fitted absorption spectrum of PLL NPs in Fig. 4(c) shows the blue and redshift in these two major peaks of the ICG molecules. The red and blue shifts in the absorption peaks indicate the possible H and J aggregation; therefore, this result confirms the encapsulation of the ICG in the aggregated form within PLL NPs. In addition, a significant broadening of the monomeric peak ~45 nm is observed in the case of PLL NPs in comparison to the free-form of the ICG. This suggests that along with aggregates, there might be a significant number of ICG molecules in the monomeric form, as well. In summary, the spectral peak shifting, broadening, and absorption reduction (as shown in Fig. 4(a)) indicate the encapsulation of ICG molecules in the aggregated form within PLL NPs. To further confirm the ICG aggregation within PLL NPs, the NIR fluorescence emission spectra of the free ICG and the PLL NPs were collected and compared. The fluorescence emission was recorded from 750 nm to 850 nm after excitation of the samples at 680 nm. The fluorescence spectrum of PLL NPs shows a decrease in fluorescence emission as compared with the free ICG, as shown in Fig. 4(d). This decrease in fluorescence emission of ICG within PLL NPs could be attributed to aggregation-caused quenching (ACQ).

### Interaction study of salt, ICG, and polymer

**Figure 5:**
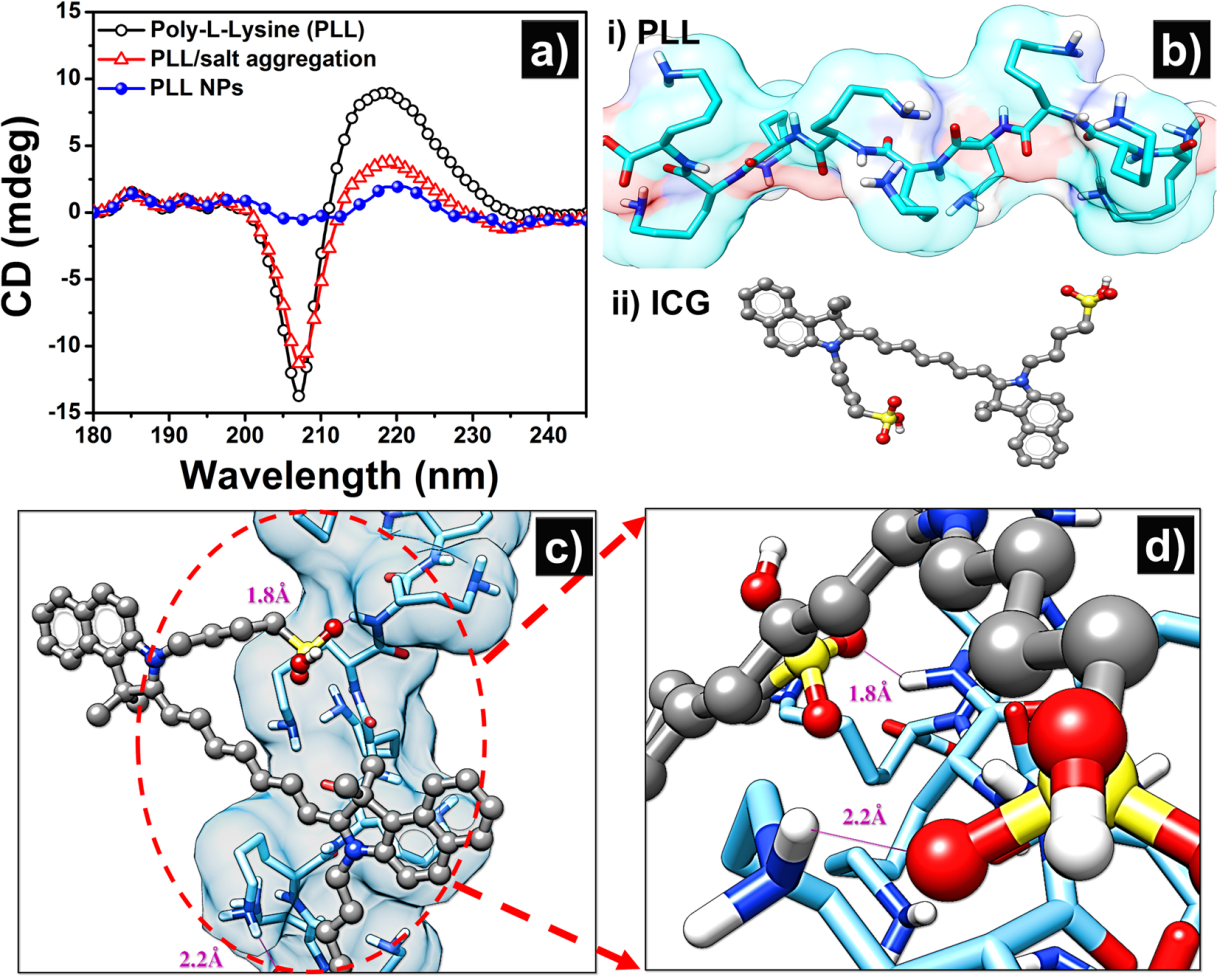
Interaction of the PLL and ICG (**a**) CD spectra of PLL and its interaction with salt and ICG (**b**) 3D structure of PLL and ICG used for docking (**c**) Complex of PLL and ICG show its favourable docking site (**d**) Docking results with two hydrogen bonds. Carbon atoms of ICG are grey, oxygen-red, hydrogen white and sulphur – yellow, carbon atoms of PLL are cyan, nitrogen is blue and hydrogen is white.

The constituents of NPs are PLL, ICG, and salts, which form nanostructure via self-assembly mechanism. Their ability to interact with each other and form a complex structure is the fundamental basis for the fabrication of these NPs (Fig. 5). We used circular dichroism (CD) spectroscopy to probe the influence of salt and ICG in PLL NPs fabrications. Figure 5(a) shows the CD spectra of aqueous PLL, PLL with a salt solution, and PLL NPs. The CD spectrum of PLL exhibits one negative band in the ultraviolet region at ~207 nm and one positive band at ~218 nm, which confirms its random coil conformation^52^. Figure 5(a) shows an increase in the band intensity at 207 nm and a decrease in band intensity at 218 nm after the addition of the salt solution, which corresponds to the interaction of PLL molecules with salts. The addition of ICG to the complex of PLL and salt further increases the intensity of the peak at ~207 nm and a reduction of the peak at ~218 nm. These changes in CD spectra confirm the formation of the nanostructure, as shown earlier by the SEM imaging. This result suggests that ICG actively interacts with PLL molecules and contributes to the formation of the stable nanostructure.

In order to further understand the role of ICG in nanostructure formation, *in-silico* studies were carried out on the PLL and ICG complexation. Docking analysis generally carried out to obtain information about the binding interactions involved during the complexation of the macromolecule with ligand^61,62^. There have been numerous experimental studies on the self-assembly process for the fabrication of NPs. However, there is rarely any report to show the *in-silico* interaction of ligand and macromolecule during the self-assembly process. In this study, PLL was used as a macromolecule, and ICG was used as a ligand for the *in-silico* studies to show the interaction between them during NP formation. This result shows that the net negative charge on ICG plays an important role in the self-assembly process and contributes to the formation of stable PLL NPs. The docking results show that there is active binding between ICG (ligand) and PLL (macromolecule). Specifically, the binding between ICG and PLL is due to the interaction between the lysine residues of PLL and sulfonate groups of ICG as shown in Fig. 5(c,d). It was found and demonstrated that the docked ligand is close to Lysine and lies within the hydrogen bonding with the sulfonate group. The distance from Lysine residue of PLL to the adjacent oxygen atom of ICG lies in the range of 1.8 Å to 2.2 Å, which is calculated by the University of California, San Francisco (UCSF) chimera Viewer. Docking studies reveals that the docked ICG makes two hydrogen bonds with Lysine, and the bond length was <2.2 Å, thus suggests a possible aggregation of ICG molecule within PLL/salt aggregates. The free energy of binding of the most populated energy cluster obtained from blind docking of the PLL using AutoDock 4.2 is found to be −ve 3.28 kcal mol^−1^ at 298 K. This study confirms the experimental findings of CD measurements of ICG-PLL complex formation. This also confirms the active participation of the ICG in the formation of PLL NPs via the self-assembly method.

### Encapsulation efficiency

As seen above, due to the possible aggregation of ICG molecules within PLL NPs, these NPs might have higher encapsulation efficiency. The encapsulation efficiency of ICG within PLL NPs was estimated using Eq. (2). This method provides a molar concentration of entrapped ICG within the PLL NPs. For the concentration estimation, PLL NPs were dissolved in DMSO, which causes the instant release of the ICG from the NPs. Following the addition of DMSO, the ICG content was quantified by measuring the absorption at 798 nm in the UV-Vis-NIR spectrophotometer. The ICG encapsulation efficiency was found to be 43.4 ± 5%.

### *In-vitro* release study

**Figure 6:**
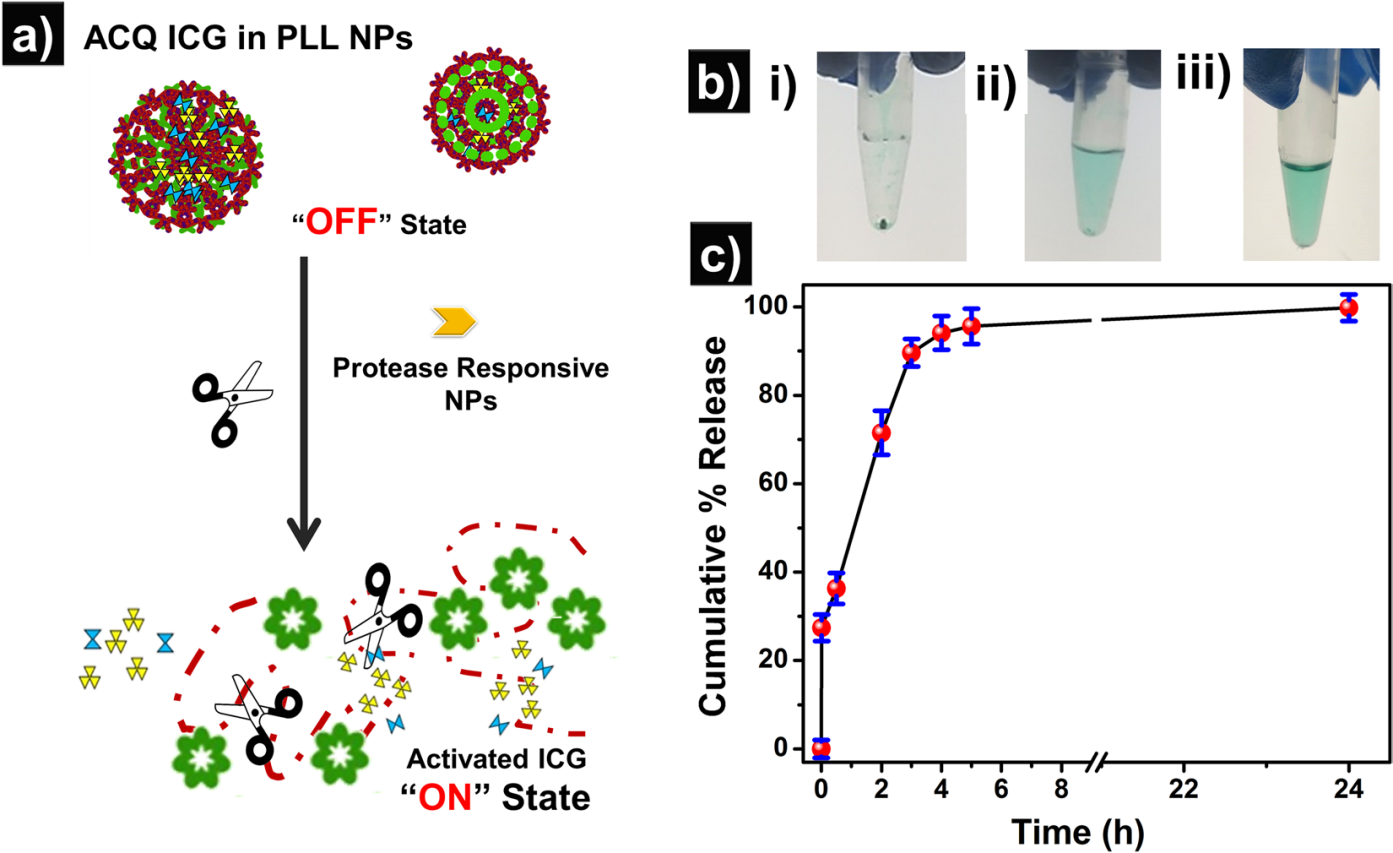
Proteolytic enzyme responsive release of ICG from PLL NPs (**a**) schematic showing the release mechanism (**b**) pictorial representation of the Eppendorf tubes after time interval (i) 5 min (ii) 4 h (iii) 24 h (**c**) cumulative *in-vitro* release study of the ICG upon incubation with trypsin at 37 °C for 24 h.

Smart nanostructure, which could release its cargo under a specific environment, are promising carriers for drug delivery^58,63^. These NPs are designed to deliver the cargo in a specific environment, such as pH, a proteolytic enzyme, *etc*. It has been shown that PLL degrades in the presence of the proteolytic enzymes^64,65^. We hypothesize that the PLL NPs are readily taken up by the cells via endocytosis and get degraded in the presence of a proteolytic enzyme in the lysosomes of the cells, which subsequently results in the release of the free-form of the ICG from NPs (Fig. 6). The schematic of a proposed mechanism for the *in-vitro* release study of the ICG from PLL NPs is shown in Fig. 6(a). As evident from the emission measurements, the fluorescence emission from PLL NPs is significantly lower than the free form of the ICG due to ACQ termed as “OFF state.” However, the hydrolysis of PLL NPs via proteolytic enzyme results in the fragments of lysine, salts, and free form of the ICG termed as activated ICG “ON state.” The released activated ICG within the cells is in monomeric form in comparison with an aggregated form within PLL NPs. Thus, the loaded ICG is protected within PLL NPs under the physiological and gets delivered to the cells. The *in-vitro* release behavior of the ICG from PLL NPs was studied after the incubation of NPs with a proteolytic enzyme, which mimics the condition of lysosomes. Under this condition, the ICG release kinetics from PLL NPs was observed and is shown in Fig. 6(b,c). Figure 6(b-(i–iii)) shows the digital picture of the Eppendorf tube showing ICG release from PLL NPs when incubated with a proteolytic enzyme (trypsin) for 5 min, 4 h, and 24 h respectively. As seen in Fig. 6(b-i), 5 min incubation of PLL NPs with proteolytic enzyme results in very less release of the ICG. After 4 h of incubation, more than 90% of ICG was released from the NPs, and a tiny NP pellet was observed. However, no pellet was observed after 24 h of incubation. Figure 6(c) shows the ICG release kinetics at different time points. As seen in Fig. 6(c), an initial abrupt release of about 38% of ICG from PLL NPs was observed within 30 mins of trypsin incubation. In the next 4 h, more than 90% of ICG was released, which confirms the enzymatic degradation property of PLL NPs. This enzymatic degradation of PLL NPs within cells would help them for a controlled release only within cells and would protect ICG while in circulation.

### Photo-stability of free ICG and PLL NPs

**Figure 7:**
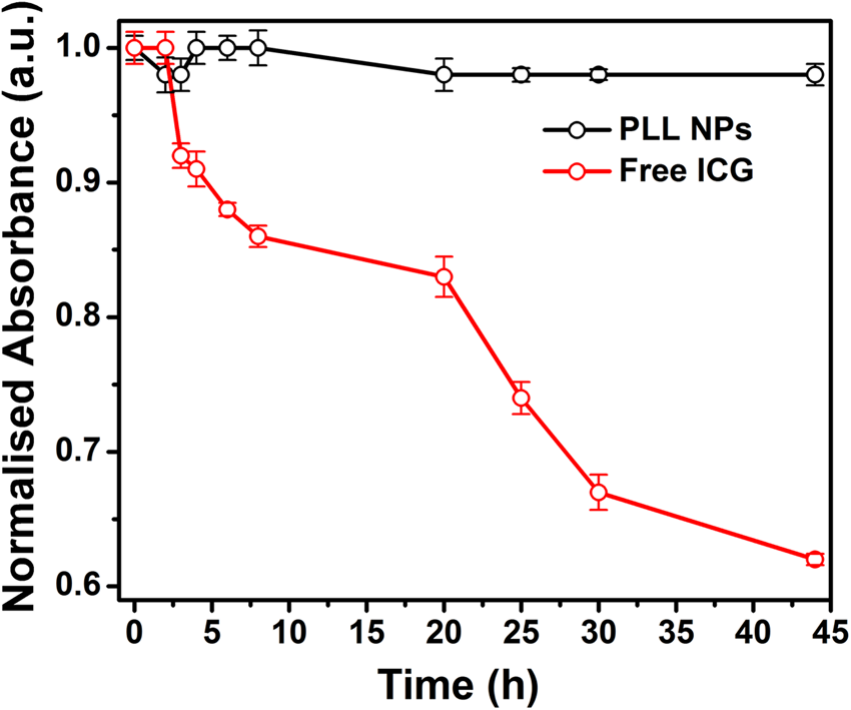
Photostability of the PLL NPs vs. free ICG in ambient light at room temperature.

Free ICG is an unstable chromophore; its optical properties get deteriorated when stored in ambient light. Therefore, for the imaging-based biomedical applications, it is important to protect free ICG from the exposure of ambient light. The nanoencapsulation of ICG might also protect it from unwanted exposure of light and maintain its optical properties. The effect of nanoencapsulation on the photostability of ICG was studied and compared with the free formulation of ICG. To study the effect of light exposure on PLL NPs and free ICG solutions, samples were exposed to ambient light at room temperature (RT) for 45 h. At each time point, the absorbance of PLL NPs and ICG was measured by the UV-Vis-NIR spectrophotometer. As shown in Fig. 7, PLL NPs are more optically stable than free ICG under ambient light exposure at RT. The free ICG sample lost ~40% optical activity within 45 h of ambient light exposure inside the laboratory setup. However, nanoencapsulated ICG lost only ~4% of its optical activity. This result suggests that the nanoencapsulation of free ICG also protects it from optical degradation.

### Cellular toxicity and uptake study

**Figure 8:**
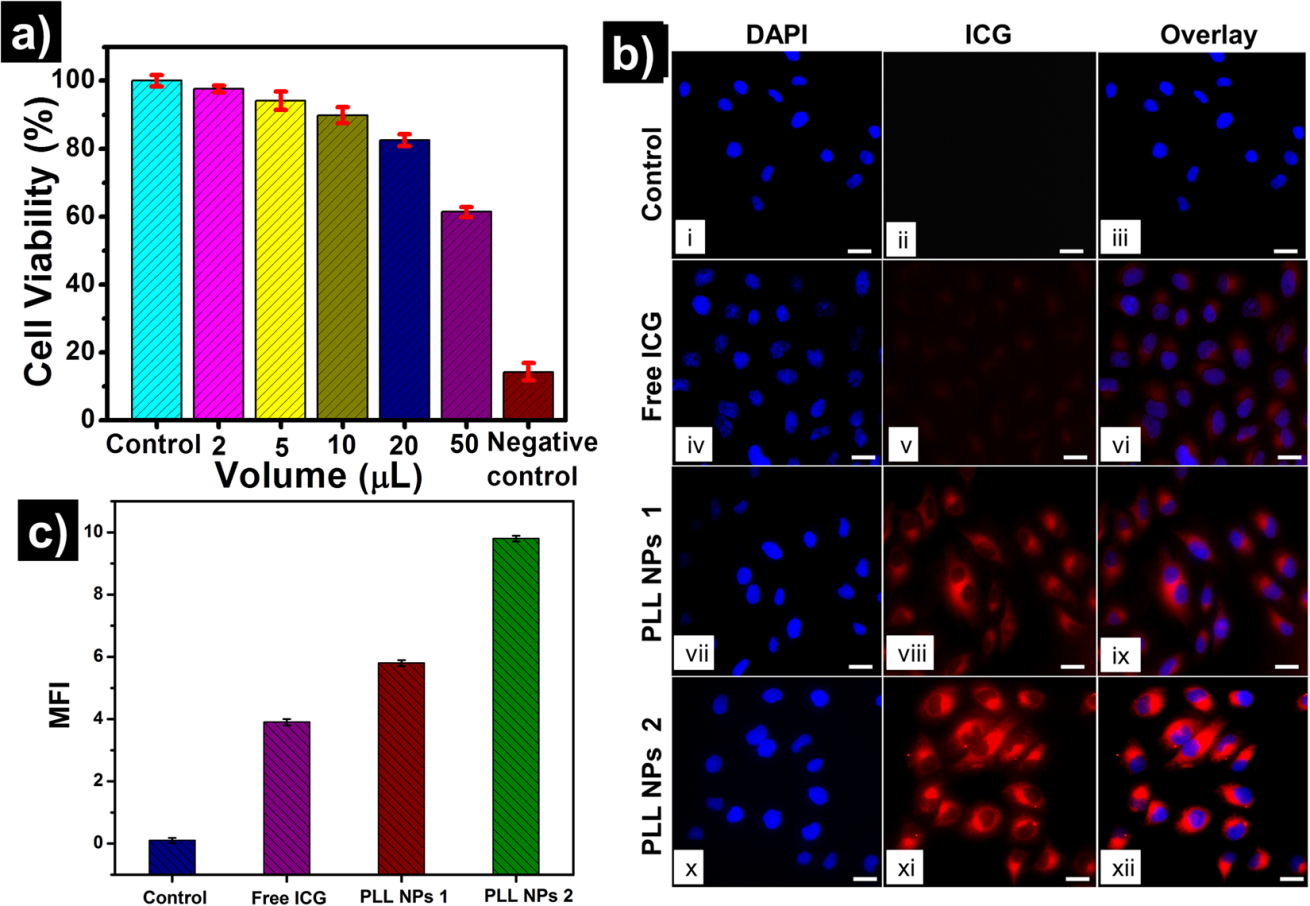
Cellular viability and NIR imaging of HeLa cells treated with free indocyanine green (ICG) and ICG encapsulated poly-l-lysine nanoparticles (PLL NPs). (**a**) The cellular viability of two different concentrations of PLL NPs with positive and negative control. (**b**) NIR imaging of HeLa cells, where (i-iii) were control cells, (iv-vi) free ICG treated cells and (vii-xii) were treated with two different concentrations of PLL NPs. The DAPI staining nuclei are denoted in blue, and ICG emission was denoted in red colour. Scale bar: 20 µm. (**c**) Fluorescence intensity quantification by IMAGE J software.

For biomedical applications, the safety of these NPs needs to be established. The *in-vitro* cytotoxicity of PLL NPs was evaluated through MTT assay, and cellular imaging was done on HeLa cells, as shown in Fig. 8. The result of cell viability experiments are shown in Fig. 8(a) where HeLa cells were incubated with different concentrations of PLL NPs for 24 h. The cells incubated with 2 μL and 5 μL concentration of PLL NPs show more than 94% viability at both the concentrations. However, the cells incubated with 10 μL and 20 μL PLL NPs were also found to be safe and showed ~89% and ~82% cellular viability respectively. A decrease in the cellular viability was observed when cells were incubated with 50 μL of PLL NPs; these cells showed ~60% cellular viability. The HeLa cells without any treatment were used as a positive control. The cells incubated with Triton x were used as a negative control and showed less than 10% cell viability. This result suggests that PLL NPs are safe to be used as a delivery vehicle, as reported for biomedical applications. Following the cell viability study, experiments were carried out to study the potential of PLL NPs for drug or contrast agent delivery applications.

For the bioimaging experiment, HeLa cells were incubated with free ICG, two concentrations of PLL NPs, and PBS (as control) for 4 h. The results of cellular imaging are shown in Fig. 8(b), where column 1 shows the nuclei of the cells stained with DAPI (blue), column 2 shows the NIR fluorescence emission from ICG (red), and column 3 shows the merged image of the channel 1 and 2. The rows represent the following treatment; the first row shows control, the second row shows free ICG, and two different concentrations of PLL NPs are shown in third and fourth rows respectively. As expected, no fluorescence emission was detected from control HeLa cells, and a weak fluorescence is observed from free ICG incubated cells. This indicates that free ICG was not readily taken up by the cells in 4 h incubation. However, significantly higher fluorescence emission was observed from PLL NPs treated cells. In addition, if the concentration of PLL NPs is increased, the higher fluorescence emission from the cells is observed.

Cellular imaging results confirm the significantly higher fluorescence emission from PLL NPs treated cells than free ICG treated cells, which shows the effectiveness of PLL NPs for NIR imaging. It is pertinent to note that fluorescence emission is emitted from the cytosol of the cells; this is possible due to the proteolytic degradation of PLL NPs in the lysosomes. To quantify the fluorescence emission from the different treatments mean fluorescence intensity (MFI) of NIR fluorescence emission from HeLa cells was calculated using IMAGE J software (shown in Fig. 8(c)). It is important to note that control cells showed no fluorescence and PLL NP treated cells showed the highest NIR fluorescence. This result supports our *in-vitro* release study which indicates that PLL NPs are efficient biological nanocarrier for delivering ICG in the presence of proteolytic enzymes. In addition to the delivery of ICG to the cells, the PLL NPs can also be used to encapsulate and deliver the therapeutic agents to the targeted cells and tissues.

## Conclusion

In summary, we have demonstrated the first-ever green chemistry-based fabrication of ICG encapsulated PLL NPs, where the presented NPs were fabricated using a self-assembly method at room temperature without using any organic or toxic solvents. Unlike conventionally fabricated NPs, the presented NPs would be free from the toxicity caused by the reagents used in fabrication, therefore they would be highly useful in *in-vivo* biomedical applications. These NPs have a spherical morphology and high encapsulation efficiency of ICG. These NPs are highly monodispersed with positive zeta potential and show no aggregation. The cellular viability studies show that more than 95% of PLL NPs treated cells were viable after 24 h of incubation. In addition, the release of the cargo was facilitated only inside the cells via proteolytic enzymatic degradation. The biocompatibility (with zero toxicity) and biodegradability of these NPs make them a suitable carrier for the drug delivery and the delivery of exogenous contrast agents. Therefore, these PLL NPs may serve as a potential nanocarrier for various theranostic applications.

## Supplementary information

Supplementary data.
