# Supplementary material for: Protease Responsive Essential Amino-Acid Based Nanocarriers for Near-Infrared Imaging
Source: Sci Rep. 2019 Dec 30;9:20334. doi: 10.1038/s41598-019-56871-4 (PMC6937316; doi:10.1038/s41598-019-56871-4)
Supplement: Supplementary file 1 — Supplementary data. [file 41598_2019_56871_MOESM1_ESM.pdf]

Supporting Information

Protease Responsive Essential Amino-Acid Based Nanocarriers for Near-Infrared Imaging

*Anshu Kumari,[1] Kalpana Kumari[1] and Sharad Gupta[1, 2]**

[1]Discipline of Biosciences and Biomedical Engineering, Indian Institute of Technology Indore, Khandwa Road, Simrol, Indore, India-453552

[2]Metallurgical Engineering and Material Science, Indian Institute of Technology Indore, Khandwa Road, Simrol, Indore, India-453552

1. Synthesis of poly-l-lysine nanoparticles (PLL NPs):

A cocktail of precooled salt solutions of trisodium citrate dihydrate 13.2 µL (0.01 M, 4 ^{o} C) and 2 µL of disodium phosphate heptahydrate (0.01 M, 4 ^{o} C) was added dropwise to 20 µL of pre-cooled PLL solution (3mg/mL, 4 ^{o} C). The whole solution was gently mixed for 10 seconds as shown in Fig. S1(a, b and c). Then, 200 µL of cooled ICG solution (645 µM, 4 ^{o} C) was added in a self-assembled colloidal mixture of PLL and salt and mixed for 10 seconds, and finally, water was added to make final volume to one mL shown In Fig. S1(d). The final mixture was aged for 30 minutes at 4 ^{o} C, and the resultant solution was immediately, differentially centrifuged at 6,500 rpm for 1 min

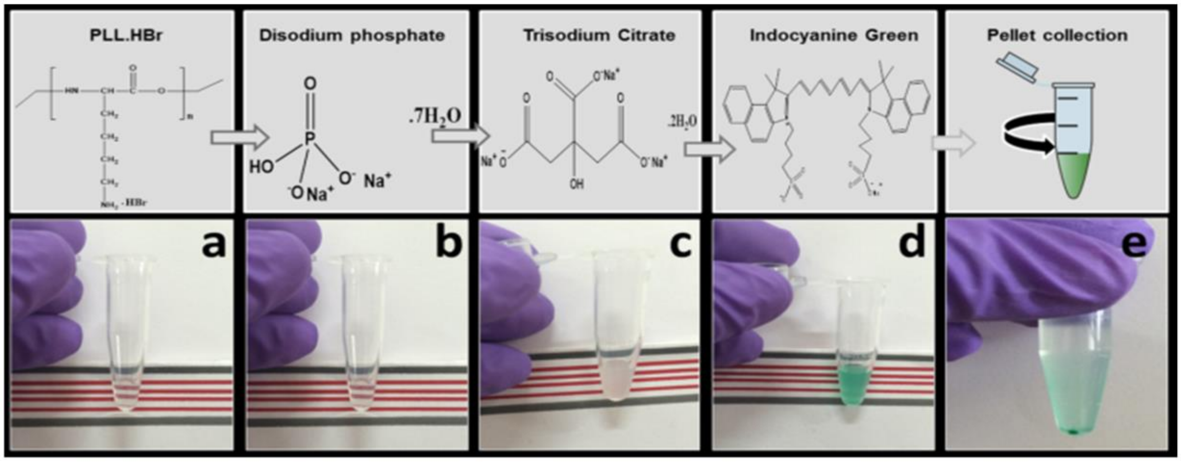

Figure S1. A Pictorial diagram illustrating the process of fabricating poly-l-lysine (PLL) nanoparticles formation. (a) Showing a clear solution of PLL in 1.5 mL Eppendorf tube. (b) Addition of disodium phosphate salt. (c) Mixing of trisodium citrate salt. (d) Pipetting 200 µL of ICG for 10 seconds (e) Image of the pellet after differential centrifugation.

followed by 6000 rpm for 30 min, and 5500 rpm for 60 min and the pellet was collected for characterization as shown in Fig. S1(e).

2. Effect of variation in pH and MCR of the solution on PLL NPs diameter:

As shown in figure S2 the variation on the particle size was visualized under FESEM after changing pH of the salt. Figure S2 (a) shows the particle formation with size ~ 250 nm but aggregation was there. In Figure S2 (b) particles are formed without aggregation with size ~190 nm. Further in Figure S2 (c and d), particles are formed with diameter of ~ 291 nm and ~ 350 nm in size.

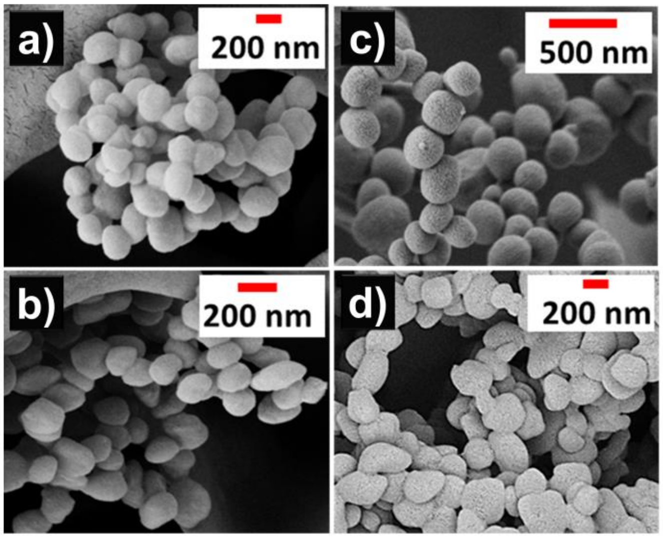

Figure S2. FESEM of the particles synthesized with varying the pH value of the salt. (a) pH 4.3 (b) 7.5 (c) 10.4 and (d) 11.6.

Further the FESEM images of the formed PLL NPs by varying the MCR of the system was shown in Figure S3. As the MCR is increased above 2 the PLL NPs size are increased as shown in Figure S3 (c-f). The best MCR is 2 where 170 nm PLL particles are formed with monodispersed particles without any aggregation. However, when MCR is 1.4 ~231 nm particles size is formed with monodispersing in nature. Further when MCR were set to be between 2 to 6 the size is increased with diameter ~1000 nm, ~1100 nm, ~ (1400 to 1600 nm) and 2200 nm respectively.

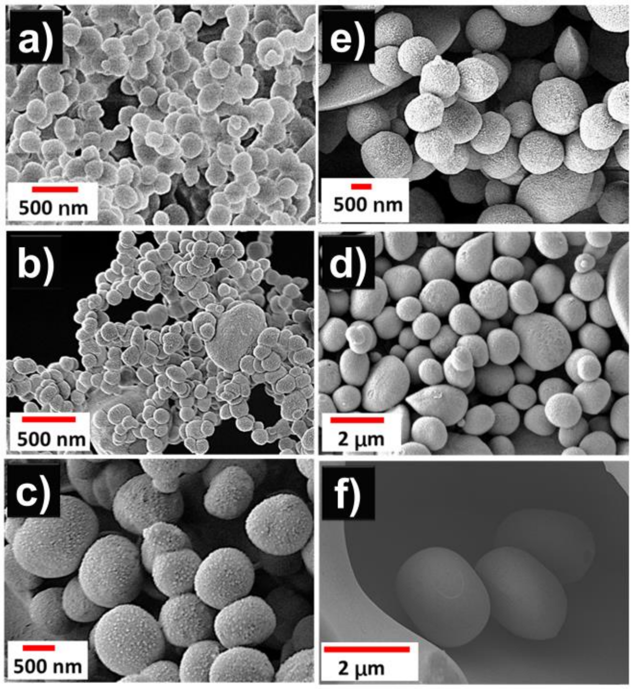

Figure S3. FESEM images of PLL NPs after varying the MCR of the system(a) MCR – 1.4 (b) MCR – 2 (c) MCR – 3 (d) MCR – 4 (e) MCR – 5 (f) MCR – 6. MCR- molar charge ratio.

3. Spectroscopic Characterization:

The absorption spectrum of free ICG was recorded by UV-Vis-NIR spectrophotometer. Calibration curve of absorbance versus different concentration of free ICG in dimethyl sulfoxide (DMSO) was plotted as shown in Figure S4.

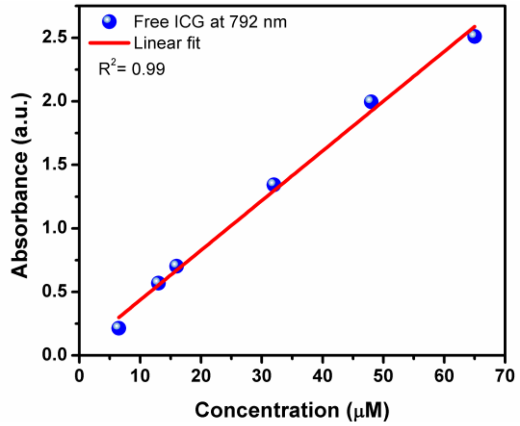

Figure S4. Calibration curve of free indocyanine green (ICG) in dimethyl sulfoxide (DMSO).

This calibration curve was used to measure the concentration of ICG in PLL NPs. For concentration measurement, PLL NPs were dissolved in DMSO which break self-assembled PLL NPs and release ICG in its free form, and its absorption was measured to find out the concentration.

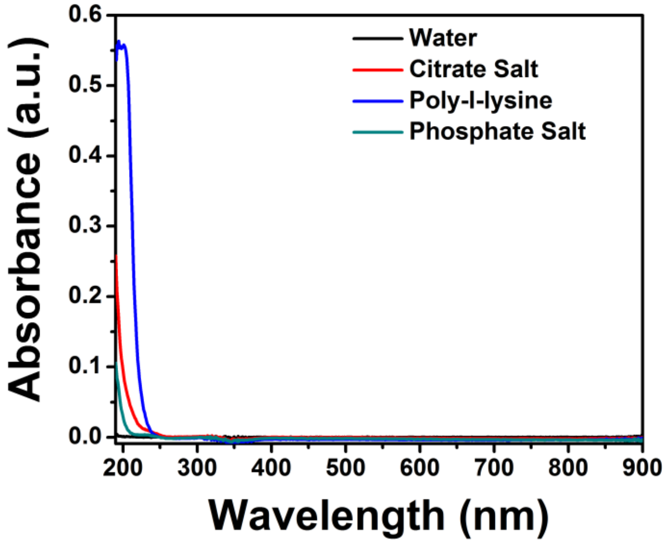

Figure S5. Absorption Spectra of poly-l-lysine disodium citrate, water and trisodium citrate salt.

All constituents used for the fabrication was PLL, citrate salt, disodium phosphate salt and water. Figure S5 shows the absorption spectra of all the constituents, except ICG, used for the fabrication of the PLL NPs. It was observed that all other constituent except ICG did not have absorption in visible to NIR wavelength range.
